# Trends in Sexual Risk Behaviors Among Hispanic/Latino Men Who Have Sex with Men — 19 Urban Areas, 2011–2017

**DOI:** 10.15585/mmwr.mm6840a2

**Published:** 2019-10-11

**Authors:** Lindsay Trujillo, Johanna Chapin-Bardales, Emilio J. German, Dafna Kanny, Cyprian Wejnert, the National HIV Behavioral Surveillance Study Group, National HIV Behavioral Surveillance Study Group, Meaghan Abrego, Alia Al-Tayyib, Bridget Anderson, Narquis Barak, Greg Bautista, Lissa Bayang, Jeremy M. Beckford, Nanette Benbow, Trista Bingham, Barbara Bolden, Kathleen A. Brady, Mary-Grace Brandt, Sarah Braunstein, Richard Burt, Rosalinda Cano, Sidney Carrillo, Jie Deng, Rose Doherty, Anna Flynn, Colin Flynn, David Forrest, Dawn Fukuda, Danielle German, Sara Glick, Vivian Griffin, Henry Godette, DeAnn Gruber, Emily Higgins, Theresa Ick, Tom Jaenicke, Antonio D. Jimenez, Salma Khuwaja, Monina Klevens, Irene Kuo, Marlene LaLota, Zaida Lopez, Yingbo Ma, Karen MacMaster, Kathryn Macomber, Manya Magnus, Melissa Marzan, Melanie Mattson, David Melton, Sharon Melville, Lisa Metsch, Vanessa Miguelino-Keasling, Maura Miminos, Sandra Miranda De León, Alan Neaigus, Willie Nixon, Chrysanthus Nnumolu, Alicia Novoa, Conall O’Cleirigh, Jenevieve Opoku, Paige Padgett, Jonathon Poe, Nikhil Prachand, H. Fisher Raymond, Hafeez Rehman, Kathleen H. Reilly, Alexis Rivera, William T. Robinson, Yadira Rolón-Colón, Kimi Sato, John-Mark Schacht, Stephanie Masiello Schuette, Ekow Kwa Sey, Shane Sheu, Jennifer Shinefeld, Mark Shpaner, Andrea Sifferman, Amber Sinclair, Lou Smith, Emma Spencer, Ashley Tate, Hanne Thiede, Mark Thrun, Jeff Todd, Veronica Tovar-Moore, Margaret Vaaler, Al Velasco, Carol-Ann Watson, Travis Wendel, Tiffany West, Jianglan White, Ralph Wilmoth, Chris Wittke, Afework Wogayehu, Marcia Wolverton, Pascale Wortley, Meagan C. Zarwell

**Affiliations:** ^1^Division of HIV/AIDS Prevention, National Center for HIV/AIDS, Viral Hepatitis, STD, and TB Prevention, CDC; ^2^Oak Ridge Institute for Science and Education, Oak Ridge, Tennessee.; Nassau and Suffolk counties, New York; Denver, Colorado; Nassau and Suffolk counties, New York; New Orleans, Louisiana; Atlanta Georgia; San Diego, California; New Orleans,; Louisiana; Chicago, Illinois; , Los Angeles,; California; Newark, New Jersey; Philadelphia, Pennsylvania; Detroit, Michigan; New York City, New York; Seattle, Washington; San Diego, California; New York City, New York; Dallas, Texas; Boston, Massachusetts; San Diego,; California; Baltimore, Maryland; Miami, Florida; Boston, Massachusetts; Baltimore, Maryland; Seattle, Washington; Detroit, Michigan; Newark, New Jersey; New Orleans,; Louisiana; Detroit, Michigan; San Francisco, California; Seattle, Washington; Chicago, Illinois; Houston, Texas; Boston, Massachusetts; Washington; D.C.; Miami, Florida; Houston, Texas; Los Angeles, California; Detroit, Michigan; Detroit, Michigan; Washington; D.C.; San Juan, Puerto Rico; Denver, Colorado; Atlanta, Georgia; Dallas, Texas; Miami, Florida; San Diego, California; Boston, Massachusetts; San Juan, Puerto Rico; New York City, New York; Miami, Florida; Philadelphia, Pennsylvania; Dallas, Texas; Boston, Massachusetts; Washington; D.C.; Houston, Texas; Dallas, Texas; Chicago, Illinois; San Francisco, California; Houston, Texas; New York City, New York; New York City, New York; New Orleans, Louisiana; , San Juan, Puerto Rico; Atlanta, Georgia; Miami, Florida; Chicago, Illinois; Los Angeles, California; Dallas, Texas; Philadelphia, Pennsylvania; Philadelphia, Pennsylvania; Philadelphia, Pennsylvania; Nassau and Suffolk counties, New York;; Nassau and Suffolk counties, New York; Miami, Florida; Nassau and Suffolk counties, New York; Seattle, Washington; Denver, Colorado; Atlanta, Georgia; San Diego, California; Dallas, Texas; San Diego, California; Nassau and Suffolk counties, New York; New York City, New York; Washington, D.C.; Atlanta, Georgia; Denver, Colorado; Boston, Massachusetts; Newark, New Jersey; Houston, Texas; Atlanta, Georgia; New Orleans, Louisiana.

Correct and consistent condom use and human immunodeficiency virus (HIV) preexposure prophylaxis (PrEP) are protective against sexual transmission of HIV (*1*,*2*). The incidence of HIV infection among Hispanic/Latino men who have sex with men (MSM) in the United States is increasing (*3*). HIV risk among Hispanic/Latino MSM differs based on their place of birth and years of U.S. residence (*4*). Data from CDC’s National HIV Behavioral Surveillance (NHBS)* for 2011–2017 were analyzed to assess changes in sexual risk behaviors among Hispanic/Latino MSM by place of birth and years of U.S. residence. Overall, condomless anal sex during the previous 12 months increased from 63% in 2011 to 74% in 2017, and PrEP use during the previous 12 months increased from 3% in 2014 to 24% in 2017. Regardless of place of birth, nearly 75% of Hispanic/Latino MSM reported condomless anal sex during 2017. However, because of PrEP use, <60% of non-U.S.–born Hispanic/Latino MSM and <50% of U.S.-born Hispanic/Latino MSM reported unprotected anal sex (condomless anal sex and no PrEP use) during 2017. Results indicate that PrEP can be a vital tool for reducing HIV transmission among Hispanic/Latino MSM, especially those who have condomless anal sex. Interventions to prevent HIV acquisition, including increasing PrEP uptake, could address cultural and linguistic needs of Hispanic/Latino MSM, as well as other barriers to prevention of HIV infection typically faced by all MSM.

In 2011, 2014, and 2017, NHBS conducted behavioral surveys and HIV testing among MSM by using venue-based sampling. The analysis was limited to eligible participants^†^ from 19 urban areas^§^ who self-identified as Hispanic/Latino, reported having sex with another man during the previous 12 months, and had an HIV-negative test result after the NHBS interview.^¶^ Participants’ place of birth was dichotomized as U.S.-born (50 states and the District of Columbia) or non-U.S.–born. Among non-U.S.–born participants, number of years of U.S. residence was used as a proxy for acculturation (i.e., language preference), with a cutoff of ≤5 years to define recent migration (*5**,**6*). Sexual risk behavior was measured by two variables: 1) condomless anal sex during the previous 12 months and 2) unprotected anal sex, defined as condomless anal sex without having taken PrEP at any time during the previous 12 months. Log-linked Poisson regression models with generalized estimating equations clustered on recruitment event and adjusted for age and region were used to estimate adjusted prevalence ratios (aPRs) and 95% confidence intervals (CIs). Differences in the trends by place of birth and years of U.S. residence were determined using score tests to obtain interaction p-values that assessed the interaction between 1) year and 2) place of birth and length of U.S. residence. Because PrEP was approved for use in 2012,** comparisons of unprotected anal sex during the previous 12 months were made only for data collected in 2014 and in 2017. Analyses were conducted using SAS (version 9.4; SAS Institute).

This analysis included 4,731 sexually active, HIV-negative Hispanic/Latino MSM interviewed during three cross-sectional data collection cycles (2011, N = 1,581; 2014, N = 1,479; and 2017, N = 1,671) in 19 urban areas. Overall during the preceding 12 months, the prevalence of condomless anal sex increased from 63% in 2011 to 74% in 2017, and the prevalence of PrEP use increased from 3% in 2014 to 24% in 2017 (Table 1). In 2017, PrEP use in the past year was reported by 283 of 1,024 (28%) U.S.-born Hispanic/Latino MSM, 87 of 457 (19%), non-U.S.–born and resided in the United States for ≥6 years, and 30 of 188 (16%) non-U.S.–born and resided in the United States for ≤5 years.

Increases in condomless anal sex were identified among Hispanic/Latino MSM who were U.S.-born (2014 versus 2011, aPR = 1.07; 95% CI = 1.01–1.15; 2017 versus 2014, aPR = 1.06; 95% CI = 1.00–1.12) and who were non-U.S.–born and resided in the United States for ≥6 years (2014 versus 2011, aPR = 1.13; 95% CI = 1.02–1.24; 2017 versus 2014, aPR = 1.10; 95% CI = 1.01–1.20) (Table 2). Temporal changes did not differ significantly across all groups (interaction p-values: 2014 versus 2011, p = 0.72; 2017 versus 2014, p = 0.37). The prevalence of unprotected anal sex decreased during 2014–2017 among all groups, with the largest decrease occurring among U.S.-born Hispanic/Latino MSM (aPR = 0.74; 95% CI = 0.68–0.80; interaction p = 0.04). Percentages of condomless anal sex were similar among all groups during 2017 (nearly 75%). Fewer U.S.-born Hispanic/Latino MSM had unprotected anal sex (49%) than did non-U.S.–born as a result of PrEP use, regardless of years of U.S. residence (≥6 years = 58%; ≤5 years = 59%) (Figure).

**FIGURE:**
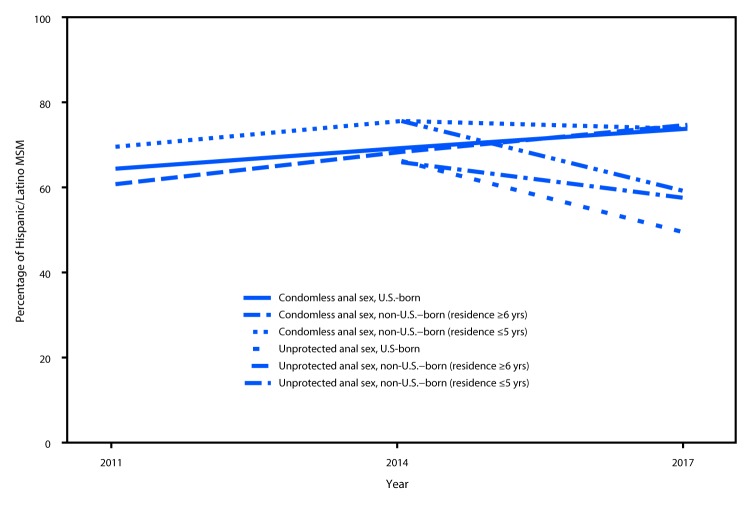
Sexual risk behaviors during the preceding 12 months among Hispanic/Latino men who have sex with men, by U.S. versus non-U.S. birth and years of U.S. residence — National HIV Behavioral Surveillance, 19 urban areas,* 2011–2017^†^ **Abbreviations:** HIV = human immunodeficiency virus; PrEP = pre-exposure prophylaxis. * Atlanta, Georgia; Baltimore, Maryland; Boston, Massachusetts; Chicago, Illinois; Dallas, Texas; Denver, Colorado; Detroit, Michigan; Houston, Texas; Los Angeles, California; Miami, Florida; Nassau and Suffolk counties, New York; New Orleans, Louisiana; New York, New York; Newark, New Jersey; Philadelphia, Pennsylvania; San Diego, California; San Francisco, California; Seattle, Washington; and District of Columbia. ^†^ Unprotected anal sex is defined as condomless anal sex without having taken PrEP at any time during the past 12 months.

## Discussion

PrEP use overall has increased among all Hispanic groups, offsetting declines in condom use. However, sexual behavioral HIV acquisition risk among Hispanic/Latino MSM differed by place of birth and years of residence in the United States. Recent residents might benefit from improved HIV prevention education and services, including access to PrEP and condoms. Further, non-U.S.–born Hispanic/Latino MSM, regardless of duration of U.S. residence, might encounter more barriers to PrEP use than do their U.S.-born counterparts (*6*). Hispanic/Latino MSM in the U.S. who prefer to use educational materials in Spanish language might be at a disadvantage for learning about PrEP and how to access it because such materials might be sparse (*7*). In addition to addressing typical barriers to PrEP use among all MSM (e.g., cost of care and stigma), HIV prevention programs and services that support Hispanic/Latino MSM, who are all facing disparities in PrEP use (*8*), might benefit from offering culturally and linguistically appropriate linkage to PrEP. CDC’s Let’s Stop HIV Together^††^ initiative has developed multiple prevention campaigns that reach MSM (e.g., Start Talking. Stop HIV^§§^ and Prescribe HIV Prevention^¶¶^) and promote PrEP awareness and use for Spanish speakers. In addition, The Latino Commission on AIDS coordinates the National Latinx AIDS Awareness Day*** observance to distribute HIV testing kits and information regarding prevention services such as PrEP though community-based organizations, health departments, and leaders among Hispanic/Latino communities In addition to other barriers to HIV prevention typically faced by all MSM (e.g., cost of care and stigma), tailoring PrEP strategies for non-U.S.–born Hispanic/Latino MSM to include improving Spanish-language materials and culturally competent patient navigation services and increasing awareness of drug assistance programs and other support services, might help reduce risk for HIV among this population.

The findings in this report are subject to at least five limitations. First, years of U.S. residence was used as a proxy for acculturation; other indicators of acculturation were unavailable for analysis. Although broadly delineating between nativity and acculturation highlights selected cultural complexities within the Hispanic/Latino MSM population, categorization into three groups remains an oversimplification of the diversity and various challenges these men face. Analysis by specific nation of birth or years of U.S. residence as a continuous variable was not possible in this study. Second, measures of PrEP use changed from 2014 to 2017; specifically, PrEP use was more narrowly defined in 2017 than in 2014.^†††^ Although PrEP use and condomless anal sex were both 12-month measures, the two might not have coincided, which might have resulted in an underestimation of the percentage of unprotected anal sex. Third, NHBS is not nationally representative, and data were not weighted to account for the complex sampling methods. Therefore, these results are not generalizable to all Hispanic/Latino MSM or to all geographic areas. Fourth, the analysis excluded interview data from San Juan, Puerto Rico, because of public health differences between Puerto Rico and the 50 states and District of Columbia (e.g., access to Medicaid and the limited number of PrEP providers). In 2017, 71% of MSM interviewed in Puerto Rico reported condomless anal sex, but only 4% reported using PrEP (*9*). Finally, data regarding self-reported behaviors, which were asked about among participants for a 12-month period, might be subject to recall error or social desirability bias, which can lead to overreporting PrEP use or underreporting condomless anal sex.

The proposed Ending the HIV Epidemic^§§§^ initiative highlights MSM and Hispanics/Latinos as priority populations for reaching to achieve national HIV prevention goals (*10*). The analyses in this report indicate that PrEP will be a crucial tool for reducing HIV transmission among Hispanic/Latino MSM. HIV prevention interventions, including linkage to PrEP, could address specific linguistic and cultural needs of Hispanic/Latino MSM and account for differences in needs by place of birth and acculturation.

**Summary**

**What is already known about this topic?**

Among Hispanic/Latino men who have sex with men (MSM), human immunodeficiency virus (HIV) infection is associated with place of birth and length of U.S. residence. Unprotected anal sex (condomless anal sex and no HIV preexposure prophylaxis [PrEP] use) increases the risk for HIV acquisition.

**What is added by this report?**

In 2017, nearly 75% of Hispanic/Latino MSM reported condomless anal sex. However, because of PrEP use, <60% of non-U.S.–born Hispanic/Latino MSM and <50% of U.S.-born Hispanic/Latino MSM reported unprotected anal sex.

**What are the implications for public health practice?**

Interventions to prevent HIV acquisition, including PrEP uptake, should address cultural and linguistic needs of Hispanic/Latino MSM.

**Table:** TABLE 1. Characteristics of Hispanic/Latino men who have sex with men — National HIV Behavioral Surveillance (NHBS), 19 urban areas,* 2011–2017^†^

Characteristic	Year, no. (%)			Chi-square p-value^§^
	2011	2014	2017	
**Place of birth, yrs of U.S. residence**				
U.S.-born	1,010 (63.9)	942 (63.7)	1,024 (61.4)	<0.001
Non–U.S.-born, ≥6	498 (31.5)	446 (30.2)	457 (27.4)	
Non–U.S.-born, ≤5	72 (4.6)	90 (6.1)	188 (11.3)	
**Age group (yrs)**				
18–24	550 (34.8)	410 (27.7)	360 (21.5)	<0.001
25–29	340 (21.5)	351 (23.7)	455 (27.2)	
30–39	384 (24.3)	428 (28.9)	510 (30.5)	
≥40	307 (19.4)	290 (19.6)	346 (20.7)	
**U.S. Census region**				
Northeast	383 (24.2)	335 (22.7)	294 (17.6)	<0.001
Midwest	126 (8.0)	103 (7.0)	95 (5.7)	
South	565 (35.7)	527 (35.6)	690 (41.3)	
West	507 (32.1)	514 (34.7)	592 (35.4)	
**Condomless anal sex in previous 12 mos**				
Yes	1,001 (63.4)	1,024 (69.3)	1,235 (74.0)	<0.001
No	577 (36.6)	453 (30.7)	433 (26.0)	
**PrEP use in previous 12 mos**				
Yes	—^¶^	45 (3.0)	400 (24.0)	<0.001
No	—^¶^	1,434 (97.0)	1,270 (76.0)	
Unprotected anal sex in previous 12 mos**				
Yes	—^¶^	985 (66.7)	876 (52.5)	<0.001
No	—^¶^	492 (33.3)	792 (47.5)	
**Total**	**1,581 (100)**	**1,479 (100)**	**1,671 (100)**	**NA**

**Abbreviations:** HIV = human immunodeficiency virus; NA = not applicable; PrEP = preexposure prophylaxis.

* NHBS collects data for 20 urban areas. Data for Puerto Rico was not included because of important public health differences (e.g., access to Medicaid and limited number of PrEP providers) between Puerto Rico and the 50 states and District of Columbia. The remaining 19 urban areas for which data was analyzed included the following (by U.S. Census region): *Northeast:* Boston, Massachusetts; Nassau and Suffolk counties, New York; New York City, New York; Newark, New Jersey; and Philadelphia, Pennsylvania; *Midwest:* Chicago, Illinois and Detroit, Michigan; *South:* Atlanta, Georgia; Baltimore, Maryland; Dallas, Texas; Houston, Texas; Miami, Florida; New Orleans, Louisiana; and Washington, DC; *West:* Denver, Colorado; Los Angeles, California; San Diego, California; San Francisco, California; and Seattle, Washington.

**^†^** Numbers might not sum to total because of missing data; percentages might not sum to 100 because of rounding.

^§^ Chi-square is testing whether the distribution of characteristics within a column changed temporally.

^¶^ Data regarding PrEP use before 2014 are unavailable.

** Defined as condomless anal sex without having taken PrEP at any time during the previous 12 months.

**TABLE 2:** Sexual risk behaviors during the previous 12 months among Hispanic/Latino men who have sex with men, by place of birth and years of U.S. residence — National HIV Behavioral Surveillance (NHBS), 19 urban areas,* 2011–2017^†^

Characteristic	Year, no. (%)			2014 vs 2011 aPR^§^ (95% CI)	p-value		2017 vs 2014 aPR^§^ (95% CI)	p-value
	2011	2014	2017					
**Condomless anal sex**								
**Overall**	**1,001 (63.4)**	**1,024 (69.3)**	**1,235 (74.0)**	**1.09 (1.02–1.18)**		**0.018**	**1.05 (0.99–1.11)**	**0.121**
**Place of birth, yrs of U.S. residence**								
U.S.-born	648 (64.3)	651 (69.3)	754 (73.8)	1.07 (1.01–1.15)	0.027		1.06 (1.00–1.12)	0.046
Non-U.S.–born, ≥6	302 (60.6)	305 (68.4)	341 (74.6)	1.13 (1.02–1.24)	0.014		1.10 (1.01–1.20)	0.025
Non-U.S.–born, ≤5	50 (69.4)	68 (75.6)	139 (73.9)	1.08 (0.90–1.30)	0.418		0.98 (0.86–1.13)	0.823
**Unprotected anal sex^¶^**								
**Overall**	—**	**985 (66.7)**	**876 (52.5)**	**—**	**—**		**0.79 (0.74–0.85)**	**<0.001**
**Place of birth, yrs of U.S. residence**								
U.S.-born	—	623 (66.3)	501 (49.1)	—	—		0.74 (0.68–0.80)	<0.001
Non-U.S.–born, ≥6	—	294 (65.9)	263 (57.5)	—	—		0.87 (0.79–0.96)	0.008
Non-U.S.–born, ≤5	—	68 (75.6)	111 (59.0)	—	—		0.78 (0.67–0.91)	0.002

**Abbreviations:** aPR = adjusted prevalence ratio; CI = confidence interval; HIV = human immunodeficiency virus; PrEP = preexposure prophylaxis.

* NHBS collects data for 20 urban areas. Data for Puerto Rico was not included because of important public health differences (e.g., access to Medicaid and limited number of PrEP providers) between Puerto Rico and the 50 states and District of Columbia. The remaining 19 urban areas for which data was analyzed included the following (by U.S. Census region): *Northeast:* Boston, Massachusetts; Nassau and Suffolk counties, New York; New York City, New York; Newark, New Jersey; and Philadelphia, Pennsylvania; *Midwest:* Chicago, Illinois and Detroit, Michigan; *South:* Atlanta, Georgia; Baltimore, Maryland; Dallas, Texas; Houston, Texas; Miami, Florida; New Orleans, Louisiana; and Washington, DC; *West:* Denver, Colorado; Los Angeles, California; San Diego, California; San Francisco, California; and Seattle, Washington.

^†^ Numbers might not sum to total because of missing data.

^§^ Models adjusted for place of birth and years of U.S. residence, age, and region and clustered on venue recruitment events.

^¶^ Defined as condomless anal sex without having taken PrEP at any time during the previous 12 months.

** Dashes indicate that data regarding PrEP use before 2014 are unavailable.

## Footnotes

* NHBS is a cross-sectional biobehavioral surveillance system conducted in urban areas with high HIV prevalence. The number of urban areas participating differs temporally.

† Eligible participants included men who were born male and self-identified as male, reported having ever had oral or anal sex with another man, resided in the interview area, were aged ≥18 years, could complete a standardized interview in English or Spanish, and provided informed consent to participate. Surveys were administered in person by trained interviewers. All participants were offered anonymous HIV testing and incentives for the interview and HIV test. The type (cash or gift card) and amount of incentive varied by urban area based on formative assessment and local policy. A typical incentive included $25 for completing the interview and $25 for providing a specimen for HIV testing.

§ The following 20 urban areas collected data during 2011–2017: Atlanta, Georgia; Baltimore, Maryland; Boston, Massachusetts; Chicago, Illinois; Dallas, Texas; Denver, Colorado; Detroit, Michigan; Houston, Texas; Los Angeles, California; Miami, Florida; Nassau and Suffolk counties, New York; New Orleans, Louisiana; New York, New York; Newark, New Jersey; Philadelphia, Pennsylvania; San Diego, California; San Francisco, California; San Juan, Puerto Rico; Seattle, Washington; District of Columbia. Participants residing and interviewed in San Juan, Puerto Rico, were excluded from the analysis because of important public health differences (e.g., access to Medicaid and limited number of PrEP providers) between Puerto Rico and the 50 states and the District of Columbia.

¶ HIV testing was performed for participants who consented. Blood specimens were collected for rapid testing in the field or laboratory-based testing. A nonreactive rapid test result was considered negative. A reactive rapid test was confirmed either with a second rapid test in the field or supplemental laboratory-based testing, typically Western blot or indirect immunofluorescence assay.

** https://aidsinfo.nih.gov/news/1254/fda-approves-first-drug-for-reducing-the-risk-of-sexually-acquired-hiv-infection.

†† https://www.cdc.gov/stophivtogether/index.html.

§§ https://www.cdc.gov/stophivtogether/campaigns/start-talking-stop-hiv/index.html.

¶¶ https://www.cdc.gov/stophivtogether/campaigns/prescribe-hiv-prevention/index.html.

*** http://nlaad.org/.

††† In 2014, participants were asked whether, in the past 12 months, they had taken anti-HIV medicines before sex because they thought it would keep them from getting HIV. In 2017, participants were asked whether, in the past 12 months, they had taken PrEP (i.e., an antiretroviral medicine such as Truvada, that is taken for months or years by a person who is HIV-negative) to reduce the risk for getting HIV.

§§§ https://www.hiv.gov/federal-response/ending-the-hiv-epidemic/overview?s_cid = ht_endinghivinternet0002.
